# Characteristics of dust mite sublingual immunotherapy-associated adverse events in the early phase

**DOI:** 10.3389/fmed.2022.1015032

**Published:** 2022-12-02

**Authors:** Ming Chen, Lin Lin, Maoxiao Yan, Chong Xu, Ruonan Chai

**Affiliations:** ^1^Department of Otolaryngology, The Second Affiliated Hospital, School of Medicine, Zhejiang University, Hangzhou, China; ^2^Department of Otorhinolaryngology-Head and Neck Surgery, Huashan Hospital of Fudan University, Shanghai, China; ^3^Department of Respiration and Anaphylaxis Treatment Center, General Hospital of North Theater Command, Shenyang, Liaoning, China

**Keywords:** sublingual immunotherapy, allergic rhinitis, adverse events, early efficacy, house dust mite

## Abstract

**Background:**

Few studies reported the characteristics of house dust mite (HDM) sublingual immunotherapy (SLIT) adverse events (AEs) during early phase treatment. The aim of this prospective study was mainly to explore the characteristics of AEs in allergic rhinitis (AR) patients during 6 months of HDM SLIT.

**Methods:**

A total of 242 patients with AR were enrolled in this study. Telephone follow-up and administration were conducted in the every week of the first month, the third month, and the sixth month of SLIT treatment. Furthermore, the early efficacy, AEs, and compliance were analyzed in our study.

**Results:**

Overall, 70.25% (170/242) of the AR patients completed the study, while 29.75% (72/242) of the AR patients failed to complete the whole 6 months of SLIT treatment process. On the whole, symptoms improved in 87.65% (149/170) of patients including 34.12% (58/170) well-controlled and 53.53% (91/170) partially controlled. The correlation analysis results showed that the treatment effect was negatively correlated with the age (*r* = −0.1614, *P* = 0.0355). The AEs mainly occurred in the first month, comprised of local rashes, gastrointestinal reactions, and itching of mouth and tongue. Subgroup analysis in the first month showed the itching of mouth and tongue, gastrointestinal reactions, fatigue, and other AEs in ≥14 years old group (14–65 years old, *n* = 42) were significant differences when compared with that in the <14 years old group (4–13 years old, *n* = 128, all *P* < 0.05). In the study, the main reasons for terminated immunotherapy were drug inaccessibility, loss of follow-up and long course of treatment.

**Conclusion:**

Patients with AR who received HDM SLIT revealed an early efficacy after 6 months, with AEs mostly occurred in the first month.

## Introduction

In the past few decades, the incidence of allergic rhinitis (AR) has increased worldwide (1). Recent epidemiological studies have also shown that AR has a high prevalence in China, resulting in high direct and indirect costs (1–5). For AR management, allergen avoidance, pharmacotherapy, allergen immunotherapy (AIT), and patient education were recommended by the World Health Organization (WHO) (6). In particular, AIT is considered as the only treatment which might change the natural course of allergic diseases (7). And as a safer treatment, sublingual immunotherapy (SLIT) is widely used in clinical (8, 9). SLIT of standardized house dust mite (HDM) has been applied in China for 16 years. A series of published clinical trials strongly demonstrated the efficacy and safety of SLIT in the treatment of HDM-induced AR in children and adults (10, 11). However, few studies researched on the characteristics of adverse events (AEs) during SLIT treatment. The objective of this study was to explore the clinical early efficacy, the characteristics of AEs, and the compliance of patients with AR after 6 months of SLIT.

## Materials and methods

### Patients

This was a prospective study. A total of 242 patients aged 4–65 years diagnosed with HDM-induced persistent AR were enrolled in the Second Affiliated Hospital Zhejiang University School of Medicine, Huashan Hospital of Fudan University, and General Hospital of North Theater Command from March to November 2020. The inclusion criteria were as follows: diagnosed with moderate to severe AR according to Allergic Rhinitis and Its Impact on Asthma (12); only sensitized to *Dermatophagoides farinae* and/or *Dermatophagoides pteronyssinus* as confirmed by a serum-specific IgE of ≥0.7 KU/L. The exclusion criteria included: the patients co-allergic to other allergens; with chronic rhinosinusitis with/without nasal polyps; who suffered from other concomitant immune system diseases or severe cardiovascular diseases; being treated with β-blockers or angiotensin-converting enzyme inhibitors; under pregnancy or lactation; and with severe psychological barriers or who were unable to understand the risks and limitations of treatment. The present study was approved by the General Hospital of North Theater Command (Y2022-007) and conducted in compliance with the Ethical Guidelines for Clinical Studies and Good Clinical Practice. All patients and their guardians were informed of the relevant information prior to their participation in the study.

### Treatment schedule

In this study, all patients were treated with standardized *D. farinae* drops (Chanllergen, Zhejiang Wolwo Bio-Pharmaceutical Co., Ltd., China) according to the manufacturer’s instructions. The treatment schedule included an up-dosing phase and a maintenance phase. The drops labeled from 1 to 5 contained proteins of 1, 10, 100, 333, and 1,000 μg/ml, respectively. The specific administration regimen was as follows: for children younger than 14 years of age, in the first 3 weeks, patients were instructed to take drops No. 1, drops No. 2, and drops No. 3, respectively. The drops were administered in the order of 1, 2, 3, 4, 6, 8, and 10 drops from day 1 to day 7. For week 4, patients were treated drops No. 4 with three drops each time, until the end of the entire treatment course. For patients over 14 years of age, usage methods and dosage in the first 5 weeks were the same as patients younger than 14 years. For week 6, patients were treated drops No. 5 with two drops each time, until the end of the entire treatment course. The drug was self-administered daily at the same time and administered sublingually for 1–3 min before swallowing. It is recommended that the patient take the first dose in hospital and be discharged after observing for at least 30 min.

### Evaluation of early efficacy

In this study, the patients’ condition at sixth months was defined as follows (13): well-controlled group defined as asymptomatic or mild symptoms that can be completely controlled with/without a few medication (use of H1 antihistamines); partial controlled group defined as mild to moderate symptoms in the case of moderate medication [use of low-dose (100–200 μg/day) intranasal corticosteroids, with/without H1 antihistamines, with/without anti-leukotrienes]; uncontrolled group defined as moderate to severe symptoms occur while taking a large amount of medication [use of high-dose (300–400 μg/day) intranasal corticosteroids, with/without H1 antihistamines, with/without anti-leukotrienes].

### Patients management and adverse events

Initial clinical education and follow-up education were carried out for all patients. Telephone follow-up and administration were conducted in the every week of the first month, the third month, and the sixth month of SLIT treatment. Furthermore, the early efficacy, AEs, and compliance were analyzed in our study.

### Statistical analysis

Statistical analysis was performed using SPSS 20.0 software (IBM Corp.). Data was expressed as mean ± standard deviation (*x* ± SD). The trend of AEs was compared to the Chi-square test. *P* < 0.05 indicated statistical significance. The Spearman bivariate analysis was performed to determine potential difference and correlation coefficient between age and treatment effect. Simple linear regression was then used to assess associations between age and treatment effect.

## Results

### Study patients

A total of 242 patients with AR were enrolled in this study. Among them, 170 patients (112 males and 58 females, mean age 11.86 ± 9.96) completed and 72 patients failed to complete the whole 6 months of treatment process.

### Early efficacy

**FIGURE 1:**
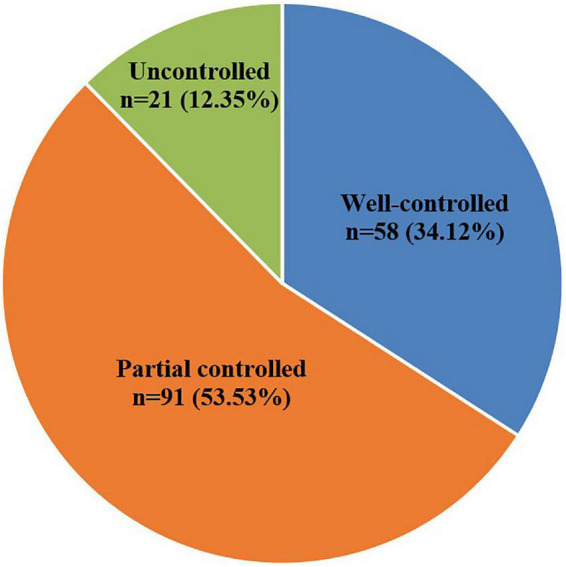
The ratio of improvement for AR patients after 6 months SLIT.

In patients who completed the study, the early efficacy improvement (well-controlled and partial controlled) rate after 6 months SLIT was 87.65%. Overall, 58 of 170 patients (34.12%) were belonged to the well-controlled group; 91 of 170 patients (53.53%) were belonged to the partially controlled group; and 21 of 170 patients (12.35%) were belonged to the uncontrolled group (Figure 1).

### Overall adverse events characteristics during the 6 month

In this study, AEs associated with SLIT included itching of mouth and tongue, local rashes, gastrointestinal reactions, fatigue, aggravating rhinitis, and other AEs. Other AEs included itchy gums and sour teeth. According to the World Allergy Organization grading system for SLIT local AEs (14), most AEs belong to grade 1 and the others belong to grade 2 in our study. No more severe AEs were reported during the entire study.

**FIGURE 2:**
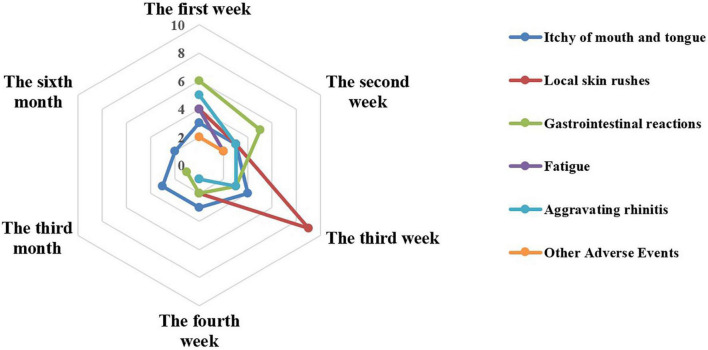
The number of adverse events at different times.

Fifty-nine patients reported 74 AEs during the entire treatment, of which 68, 4, and 2 AEs were reported in the first, third, and sixth months of treatment, respectively (Figure 2). In detail, the top three were local rashes, gastrointestinal reactions, and itching of mouth and tongue in the first month of SLIT treatment and the main symptom was itching of the mouth and tongue in the third and sixth months. Obviously, the incidences of AEs in the third and sixth months were significantly lower than in the first month (*P* < 0.001).

In the first month, 24, 18, 17, and 9 AEs were reported in the first week, the second week, the third week, and the last week, separately. And the AEs reported in the different weeks showed different characteristics for the most common AEs occurred differently. In the first week, the top three were gastrointestinal reactions (six times), aggravating rhinitis (five times), and local rashes (four times). While in the second week, five gastrointestinal reactions, three local rashes, and three itching of mouth and tongue were graded in the top three. In the following, a similar situation was eight local rashes, three itching of mouth and tongue, and three gastrointestinal reactions during the third week. At the last week, the top three included three itching of mouth and tongue, two local rashes, and two gastrointestinal reactions.

### Adverse events of different age groups in the first month

**FIGURE 3:**
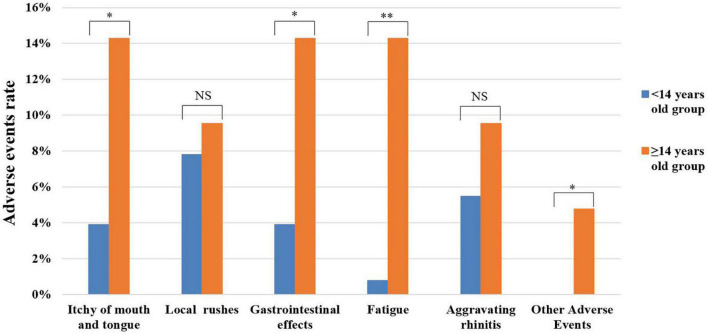
Analysis of adverse events in different age groups during the first month. NS, no significance; *P < 0.005; **P < 0.001.

Patients were divided according to age with different SLIT schedule into <14 years old group (4–13 years old, *n* = 128) and ≥14 years old group (14–65 years old, *n* = 42), and the incidence of AEs during the first month in the two groups was analyzed. In this study, the results showed that the <14 years old group was mainly characterized by local rashes, aggravating rhinitis, and gastrointestinal reactions, while the ≥14 years old group was mostly affected by gastrointestinal reactions, itching of mouth and tongue, and fatigue. We found that the incidence of AEs in ≥14 years old group was higher than that in the <14 years old group, but there were significant differences only in itching of mouth and tongue, gastrointestinal reactions, fatigue, and other AEs (all *P* < 0.05) (Figure 3).

### Correlation between age and clinical effect

**FIGURE 4:**
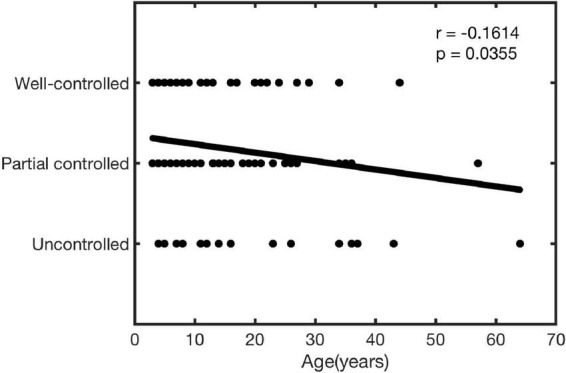
Analysis of correlation between age and clinical effect.

In this study, the correlation between age and the effects of treatment was analyzed. As shown in Figure 4, age was significantly negatively correlated with the treatment effect (*r* = −0.1614, *P* = 0.0355).

### Compliance analysis

Reasons for discontinuation of SLIT by patients were divided into seven categories: drug inaccessibility, loss of follow-up, long course, improvement of symptoms, aggravating rhinitis, catching a cold, and other reasons. Drug inaccessibility referred that the patients unable to refill their medicines because of the lockdown during the COVID-19 pandemic. Reasons for loss of follow-up included telephone number error, telephone downtime, or lost contact due to a change in private phone number. The long course included patients who cited the high frequency of drug use or long-term inability to persist as their primary reasons. Those citing improvement of symptoms believed their symptoms were alleviated and found no reason to continue treatment. Other reasons included patients who got pregnant or undergo surgery.

**FIGURE 5:**
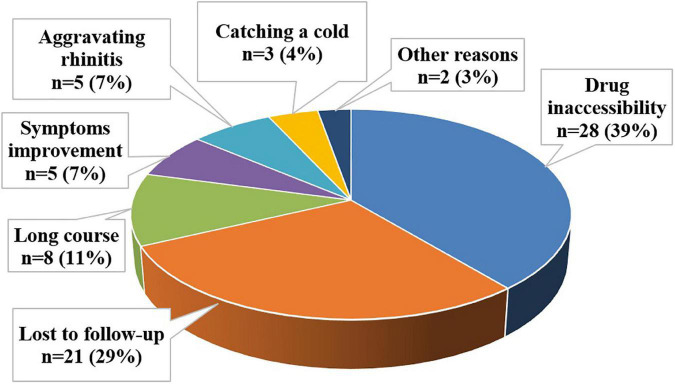
Reasons for discontinuation of immunotherapy.

In our study, the most commonly cited reason for premature cessation of SLIT was drug inaccessibility (39%). The remaining reasons leading to poor compliance were lost to follow-up (29%), the long course (11%), improvement of symptoms (7%), aggravating rhinitis (7%), catching a cold (4%), and other reasons (3%) (Figure 5).

## Discussion

A series of published clinical trials have strongly demonstrated the efficacy of SLIT on the treatment (11, 15–23), with early efficacy, sustained efficacy, long-term efficacy, and preventive efficacy (17, 22–25). SLIT has been recommended as the first-line treatment for AR (7, 11, 14, 15, 20). One research study reported that the incidence of AR in well-controlled, partially controlled and uncontrolled groups was 43.1, 32.8, and 24.1%, respectively (13). Similar results were observed in our study, the incidence of AR in the well-controlled, partially controlled, and uncontrolled groups was 34.12, 53.53, and 12.35%, resulting in an overall 87.65% improvement. This study further confirmed the early efficacy of SLIT treatment.

Sublingual immunotherapy was considered a relatively safe and well-tolerated treatment option, as it elicited fewer and milder AEs (9, 20, 26–28). Until now, most reported studies just described the overall situation of AEs. The most reported common AEs occurred during the first to the second week of SLIT treatment (8, 29, 30) and the common AEs mainly include sublingual itching, redness, or gastrointestinal reactions (11). Therefore, we further observed the differences of AEs at different time points in the early phase of treatment in this research. The relevant results showed that AEs occurred more frequently in the ≥14 years old group than the <14 years old group during the first month. In our view, two reasons might lead to this result: on the one hand, there was difference in sample size between the two groups (<14 years old group: 128, ≥14 years old group: 42); on the other hand, younger children might have inaccurate descriptions of AEs. In addition, the relationship between age and the clinical effect of SLIT was explored for the first time in this article. The results showed that age was negatively correlated with the effect of SLIT, prompting a better curative effect when they begin as soon as possible. Of course, more studies are needed to confirm this result.

It is generally considered that AIT should last for 3–5 years, and patient compliance is one of the key factors to ensure good therapeutic effect. A total of 72 people dropped out of this study. One of the main reasons was drug inaccessibility. During the COVID-19 pandemic, the Chinese government adopted strict and effective measures to control the pandemic, and the policy of closing cities/villages made it hard for patients to go to hospitals to refill their medicines. Therefore, increasing online prescription and doctor-patient communication during the COVID-19 pandemic were essential (31). At present, the problem that is being solved by an online medical platform called Easymedicare^1^ from a technology company. In addition, loss of follow-up, long course, improvement of symptoms, and aggravating rhinitis were also critical reasons leading to the poor compliance of patients. Enhancing patients’ education, both of initial clinical education and follow-up education, might be an effective solution to help patients better understand the causes of the disease, the optional treatment, and the relative characteristics (32). Furthermore, a published study reported that 6 months was recommended as the standard length for the first prescription, which could significantly improve the compliance of AR patients with SLIT (33).

## Conclusion

Patients with AR who received HDM SLIT revealed an early efficacy after 6 months, with mostly AEs occurred in the up-dosing phase, of which the top three were local rashes, gastrointestinal reactions, and itching of mouth and tongue. Moreover, the correlation analysis between age and the clinical effect of SLIT prompted the earlier of treatment leading to the better of effect. Of course, more studies are needed to confirm these results.

## Data availability statement

The datasets generated during the current study are available from the corresponding author on reasonable request.

## Ethics statement

This study was approved by the General Hospital of North Theater Command (Y2022-007) and conducted in compliance with the Ethical Guidelines for Clinical Studies and Good Clinical Practice. All patients and their guardians were informed of the relevant information prior to their participation in the study. Written informed consent to participate in this study was provided by the participants or their legal guardian/next of kin.

## Author contributions

MC designed the study. MC, LL, and RC examined the patients and wrote the manuscript. MY and CX collected the data and did statistical analysis. All authors contributed to the article and approved the submitted version.

## Footnotes

1
http://www.easymedicare.com.cn/
